# Electronic Properties of Gold(I) Compounds Relevance to Chemical Reactions

**DOI:** 10.1155/MBD.1999.223

**Published:** 1999

**Authors:** John P. Fackler, Zerihun Assefa, Jennifer M. Forward, Tiffany A. Grant

**Affiliations:** Department of Chemistry and Center for the Laboratory for Molecular Structure and Bonding Texas A&M University College Station TX 77843-3012 USA


### ELECTRONIC PROPERTIES OF GOLD(I) COMPOUNDS - RELEVANCE TO CHEMICAL REACTIONS

John P. Fackler, Jr*., Zerihun Assefa, Jennifer M. Forward, and Tiffany A. Grant

Department of Chemistry and Center for the Laboratory for Molecular Structure and Bonding, Texas A&M University, College Station, TX 77843-3012, USA

### ABSTRACT
The chemical reactivity of Au(I) compounds is related to their HOMO-LUMO energy separation. For coordinately unsaturated complexes of Au(I), the excited state (HOMO-LUMO separation) is only about 2 eV above the ground state. Many Au(I) complexes such as $[Au(TPPTS)_3]^{8-}$and $[Au(TPA)_3]^+$ show visible luminescence from this state even in water solution. Quenching of the phosphorescence relates to the rate of reaction of the substrate with the gold complex. Dioxygen and radicals such as NO rapidly react with the Au(I) to quench the excited state, as do halocarbons capable of electron and/or atom transfer. Although Corey and Khan suggested that a plausible mechanism for gold drug behavior in treatment for rheumatoid arthritis, RA, might be quenching of singlet oxygen, our work demonstrates that NO and $O_2^-$ (which leads to singlet oxygen) each react rapidly to quench emission from Au(I). Drug action may relate to the prevention of build-up in cells by Au(I) of reactive, cytotoxic peroxynitrite, $O_2NO^-$ , formed quickly from $O_2^-$ and NO in cells.

Fig. 1. Sketches of the complexes $[Au(TPA)_3]^+$ and $[Au(TPPTS)_3]^{8-}$

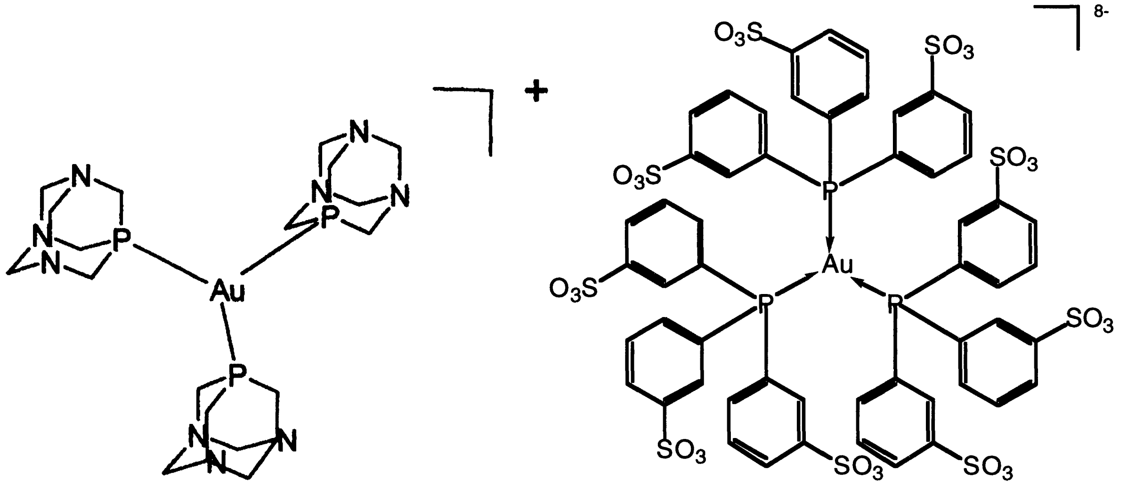

### INTRODUCTION
Commonly found oxidation states for gold include Au(0), Au(I), Au(III) and dinuclear metal-metal bonded Au(II).[1] For the oxidation state important with gold drugs, Au(I), linear two coordination, trigonal planar three coordination and tetrahedral four coordination are observed, although the first crystallographic studies had suggested that $AuL_4$ geometries were distorted from tetrahedral. With small ligands such as TPA, Figure 1, this is not the case and nearly regular tetrahedral geometries[2] have been observed, Figure 2. The electronegativity of gold is 2.1 on the Pauling scale[3], slightly less than that of C, 2.5, and I, 2.5. Gold bonds strongly to these elements in the Au(I) oxidation state.

The presence of low-lying excited states in complexes enhances their reactivity. If the LUMO of a system is not readily accessible, the compound is rather inert to chemical processes such as nucleophilic or electrophilic attack or oxidative addition. With dinuclear gold(I) compounds, the excited state can be stabilized by metal-metal interactions, promoting the trans-annular oxidative-addition of small molecules across the metal-metal bond which is formed in the product.[4] Gold-gold interactions are commonly found with Au(I) complexes. The aurophilic interaction that occurs between Au(I) centers has been estimated to have an energy of from 6 - 10 Kcal/mol in elegant work of Schmibaur.[5] Hence Au(I)-Au(I) interactions can be expected to influence reactions, even in solution.

Fig. 2. Thermal ellipsoid plot (50% probability) of $[Au(TPA)_4]Cl\cdot 6H_2O$, 2.384Å, P-Au-P angle 109.5°

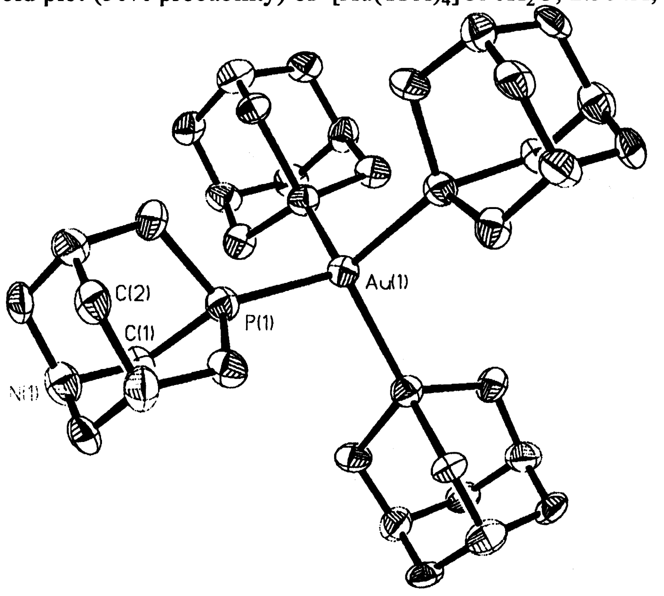

**LUMINESCENT MATERIALS**

The lowest lying excited state in gold(I) compounds often demonstrates a visible luminescence when the compounds is irradiated with UV light. This lowest lying state generally arises from the $^3D_3$ of the metal ion which is at 15,039 $cm^{-1}$ above the ground state in the gaseous ion[6] (Figure 3). Luminescence usually is observed at wavelengths around 500-700 nm.

Fig. 3. Electronic Energy Levels of Au(I) in the Gas Phase.

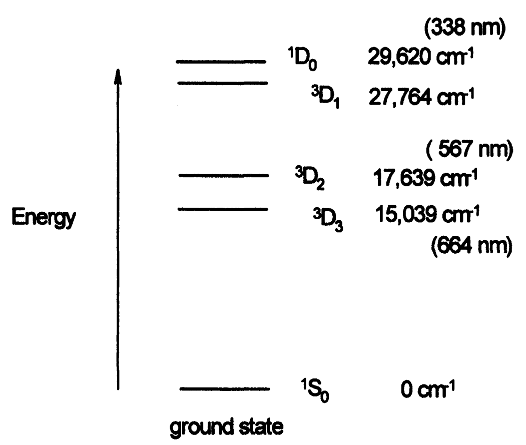

For the free ion, this transition is at 664 nm. Bonding of ligands to the Au(I) can shift the emission or completely quench it. However, for the emission to be observed as a phosphorescence, the transition out of the excited state to the ground state must be slow (forbidden). Since spin-orbit effects are large in gold, the spin "forbidden" character of the emitting state probably is less important than the orbital forbiddance in determining whether or not the state will be populated and the emission observed. With linear molecules producing s,p, and $d_{z2}$ mixing in the HOMO of $\Sigma'^+$ symmetry, the LUMO is formed largely from $p_x$,$p_y$ of $\pi$ symmetry.[7] There is no orbital forbiddance to the transitions. Phosphorescence generally is not observed. This is also true for four coordinate tetrahedral complexes in which all three valence p orbitals are involved in bonding, making the HOMO $^1T_2$ and the LUMO also $^1T_2$ symmetry. The transitions between the ground and excited states are symmetry (orbitally) allowed. Luminescence is not observed with these complexes. With planar, three coordinate complexes, the LUMO becomes mainly non-bonding $p_z$, that is orthogonal (perpendicular to the plane of the ligands) to the HOMO and does not mix with it. Transitions back to the ground state, **e**' ($d_{x2-y2}$,$d_{xy}$) are orbitally forbidden. Population of this excited state, **a**" ($p_z$)

presumably arises since it is at the lowest energy in the excited state manifold. The excitation from the $sp^2$ orbital to the mainly $a_1'$ (s) character, antibonding orbital presumably is the origin of the allowed UV transition near 340 nm.[8]

Systems with extended metal chains may emit at very low energies in the red.[9,10] Phosphine complexes and complexes with "hard" ligands show emission nearer to 500 nm. With "softer" ligands such as $Br^-$, or $I^-$ or sulfur bonded ligands, orbital mixing in the ground state produces Ligand to Metal Charge Transfer, LMCT, transitions in this region of the visible spectrum[11] and can even show multiple state emission when metal centered transitions are involved as well.[10] Figure 4 is a sketch of the types of Au(I) complexes which appear to show emission spectra. Some unusual examples have been reported recently such as the "solvoluminescent" trinuclear complex $[Au(I)(RN{=}COR)]_3$ of Balch,[12] the luminescent Au(I) dithiocarbamates of Eisenberg[13] and some linear chain complexes of TPA, $[(TPA)_2Au][Au(CN)_2]$, which only luminesce after the crystals are powdered.[14]

### LUMINESCENCE AND REACTIVITY

The connection between the chemical reactivity of a gold(I) compound and luminescence comes about in two ways. As stated above, reaction chemistry is associated with the HOMO-LUMO separation in the system. If the HOMO is energetically accessible, reactions can take place more readily either by promotion of electron density into the LUMO from the HOMO, or by nucleophilic interaction of solvent or other reactants with the LUMO. Luminescent compounds in which the latter reaction takes place lose their luminescence. Small molecules such as NO or $O_2^-$, for example, quench the luminescence of three coordinate Au(I) complexes. Electron transfer and atom transfer reactions with small electrophilic molecules such as $CCl_4$ or allylbromide can quench luminescence since the excited state is a good reductant.

Fig. 4. Types of gold(I) compounds giving visible luminescence

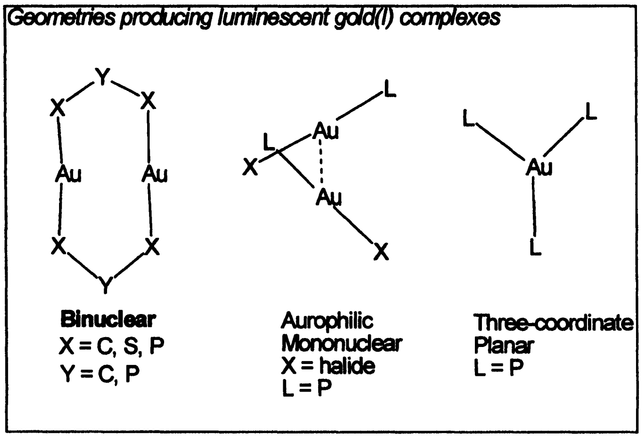

With regard to gold drug action, Corey and Khan demonstrated[15] that singlet oxygen, ($O_2$ $^1\Delta_g$), is also quenched (converted to the ground state) by gold drugs such as Auranofin at rates of the order of $10^7 M^{-1} sec^{-1}$. Singlet oxygen presumably is produced in cells of RA patients from superoxide, $O_2^-$, patients who have a deficiency of *superoxide dismutase.*[16] Heavy metals such as gold promote intersystem crossing of excited spin states and iodide has been used successfully to enhance room temperature phosphorescence for analytical purposes.[17] It is not surprising then that Au(I) drugs quench singlet oxygen ($O_2$ $^1\Delta_g$).

While gold clearly shortens the lifetime of singlet oxygen, there are generally other chemicals in a cell that can quench singlet oxygen, and sulfides are known to form sulfoxides and sulfones with this excited state of dioxygen.[18] Singlet oxygen is relatively unreactive toward saturated organics, although it does degrade olefinic polymers.[19] Reactivity with an olefin such as stilbene in minimal and the lifetime of the singlet oxygen in $CS_2$ is only ~45 ms. Thus it is uncertain how important the quenching of singlet oxygen might be by gold drugs since the species is not long lived nor highly reactive. *It is more likely that the gold drugs react with something else in the cell preventing cell damage.*

Recently it has been discovered that peroxynitrite ($ONOO^-$) is a major cytotoxic agent.[20] Since this species lives long enough to cross lipid membranes, preventing its formation or destroying it when formed seems like a reasonable postulate for gold drug action in cells. To date no studies have been reported of Au(I) compounds with peroxynitrite. However, work in our laboratory has shown that NO, $O_2$ and $O_2^-$ interact rapidly with Au(I) complexes, including $[Au(TPPTS)_3]^{8-}$ in water. Furthermore, studies of the quenching of the luminescence of this anion by small electron transfer reagents has been demonstrated[21] to be fast, k = $10^8$-$10^9$ $M^{-1}s^{-1}$. Thus it seems very reasonable that gold(I) drugs for RA inhibit formation of peroxynitrite as well as remove it when formed, by subsequent electron transfer reduction, a point that needs testing.

### PROPERTIES OF $[Au(TPPTS)_3]^{8-}$

A few years ago we demonstrated that phosphine complexes of Au(I) could be luminescent in water.[22] This work showed that Au(I) species react with $TPPTS^{3-}$ to form phosphine complexes which luminesce when the ratio of phosphine to Au(I) is 3:1. Although no structure could be obtained at the time, it was assumed that the luminescent species was the 3-coordinate complex, $[Au(TPPTS)_3]^{8-}$. The phosphine is too large to allow the formation of four coordinate complexes, as found with the TPA ligand. However, it was uncertain whether or not small molecules might interact with the Au(I). Furthermore, it was desirable to make certain that water itself did not bond to the metal ion when the ion is coordinated to phosphines. Thus various studies were performed of the properties of this ion in water.[21]

Fig. 5. Variable temperature $^{31}P$ NMR spectrum of $Na_8[Au(TPPTS)_3]$ in $CD_3OD$. Temperature is decreased from 20°C in bottom spectrum to -60° in top spectrum

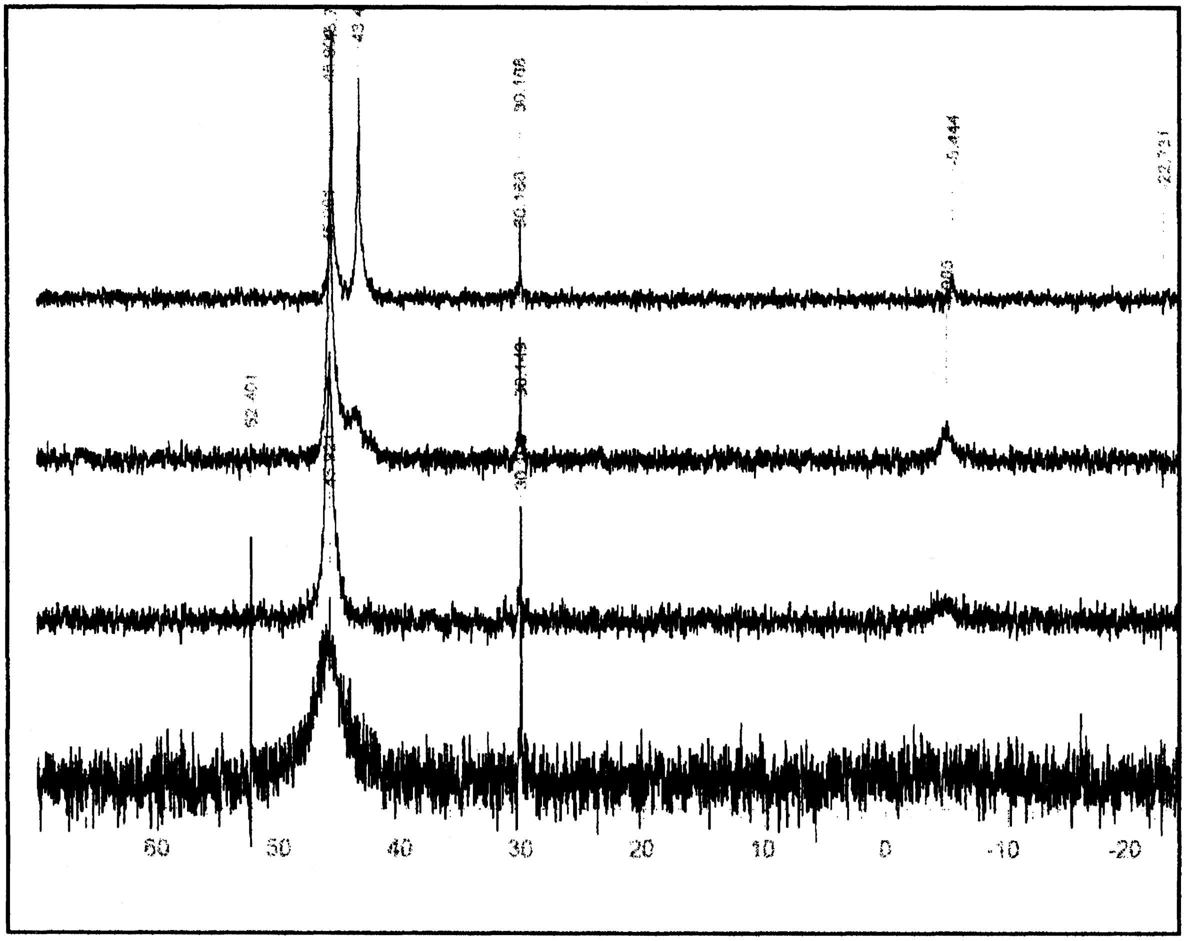

### EXPERIMENTAL STUDIES AND RESULTS

First, it was demonstrated that luminescence of the gold complex is associated with the three coordinate species. This was accomplished by careful $^{31}P$ NMR studies in concert with luminescence measurements. Using $Me_2SAuCl$ as the starting material, addition of three equivalents of $Na_3TPPTS$ in $CH_2Cl_2/H_2O$ produces a solution which upon evaporation gives a luminescent solid, unfortunately not sufficiently crystalline for structural analysis. The solid shows a $^{31}P$ NMR signal at 43 ppm. Dissolution of this complex in de-oxygenated water gives a solution that luminesces at 513 nm at 298K. If the complex is dissolved in 0.5 M NaCl, the relative emission intensity increases by a factor of 7 and the NMR line width decreases by a factor of 2. Since it had been demonstrated previously that addition or low dielectric constant solvents such as acetone of alcohols to the phosphine complex in solution reduces the luminescence intensity, this observation was consistent with the formation of a reduced coordination about the gold. The salt solution increases the dielectric constant of the solvent, apparently stabilizing the three coordinate species. The smaller NMR line width is consistent with the reduced rate of exchange of coordinated with uncoordinated phosphine. As shown in Figure 5, the broad signal at 45.5 ppm at room temperature, splits into two signals at 60°C, corresponding to the $AuL_2$ and $AuL_3$ complexes, with the latter at 43.5 ppm. The free TPPTS can be observed in the low temperature spectrum at -5.2 ppm. From the line broadening at 200 MHZ of a salt free solution, the phosphine exchange rate is estimated to be faster than 8 X $10^7$ sec.

The luminescence of the three coordinate phosphine complex in water suggested that the singlet to triplet forbidden transition might also be observed in absorption if a high concentration of the complex could be obtained. A broad absorption band indeed was observed at about 600 nm in the visible spectrum of the complex. Experimental difficulties measuring concentrations with this hygroscopic solid prevented an accurate measurement but the molar extinction coefficient for the broad band that has a width at half height of about 3,300 $cm^{-1}$ has a value between 0.5 and 1.0. Since this transition corresponds to the $^1S \rightarrow {}^3D_3$ transition of the Au(I), Figure 3, it is spin and orbitally forbidden, Furthermore, in the $D_{3h}$ symmetry of the three coordinate $AuL_3$ complex, it is symmetry forbidden also. Thus it is not surprising that it is very weak and has not been observed previously.

Fig. 6. Contents of the unit cell of $Cs_8[Au(TPPTS)_3 \cdot 5.25H_2O$, showing the complex network of bonds between the $SO_3^-$, $Cs^+$ ions and $H_2O$ molecules

Finally, the most convincing evidence that the three coordinate $AuL_3$ complex is the emitting species comes from a detailed structural characterization of the cesium salt. While the details of the crystallographic investigation will be reported elsewhere, crystals of the salt, $Cs_8[Au(TPPTS)_3]\bullet 5.25\ H_2O$, scattered x-rays sufficiently well that a data set could be obtained using a Siemans CCD SMART diffractometer at Harvard Univ. Refinement was good with $R_1 = 0.0659$.[23] As can be seen in Figure 6, a drawing of the unit cell, the Au(I) atoms are three coordinate by phosphine coordination with the hydrated Cs ions interacting with the sulfonate moieties by H-bonding. The Au-P distance ranges from 2.374 - 2.417Å in the structure, with P-Au-P angles of 128.4, 119.8 and 111.8 deg.

Figure 7, a space filling model of the structure, clearly illustrates the difficulty that large molecules would have in reaching the Au(I) center. The hydrophobic nature of this center presumably explains the inability of water molecules to quench the luminescence of the species, although this luminescence is quenched by TPA and by oxygen and other small molecules capable of electron transfer.

solvents such as acetone of alcohols to the phosphine complex in solution reduces the luminescence intensity, this observation was consistent with the formation of a reduced coordination about the gold. The salt solution increases the dielectric constant of the solvent, apparently stabilizing the three coordinate species. The smaller NMR line width is consistent with the reduced rate of exchange of coordinated with uncoordinated phosphine. As shown in Figure 5, the broad signal at 45.5 ppm at room temperature, splits into two signals at 60°C, corresponding to the $AuL_2$ and $AuL_3$ complexes, with the latter at 43.5 ppm. The free TPPTS can be observed in the low temperature spectrum at -5.2 ppm. From the line broadening at 200 MHZ of a salt free solution, the phosphine exchange rate is estimated to be faster than $8 \times 10^7$ sec.

The luminescence of the three coordinate phosphine complex in water suggested that the singlet to triplet forbidden transition might also be observed in absorption if a high concentration of the complex could be obtained. A broad absorption band indeed was observed at about 600 nm in the visible spectrum of the complex. Experimental difficulties measuring concentrations with this hygroscopic solid prevented an accurate measurement but the molar extinction coefficient for the broad band that has a width at half height of about 3,300 $cm^{-1}$ has a value between 0.5 and 1.0. Since this transition corresponds to the $^1S \rightarrow {}^3D_3$ transition of the Au(I), Figure 3, it is spin and orbitally forbidden, Furthermore, in the $D_{3h}$ symmetry of the three coordinate $AuL_3$ complex, it is symmetry forbidden also. Thus it is not surprising that it is very weak and has not been observed previously.

Fig. 6. Contents of the unit cell of $Cs_8[Au(TPPTS)_3 \cdot 5.25H_2O$, showing the complex network of bonds between the $SO_3^-$, $Cs^+$ ions and $H_2O$ molecules

Finally, the most convincing evidence that the three coordinate $AuL_3$ complex is the emitting species comes from a detailed structural characterization of the cesium salt. While the details of the crystallographic investigation will be reported elsewhere, crystals of the salt, $Cs_8[Au(TPPTS)_3]\bullet 5.25\ H_2O$, scattered x-rays sufficiently well that a data set could be obtained using a Siemans CCD SMART diffractometer at Harvard Univ. Refinement was good with $R_1 = 0.0659$.[23] As can be seen in Figure 6, a drawing of the unit cell, the Au(I) atoms are three coordinate by phosphine coordination with the hydrated Cs ions interacting with the sulfonate moieties by H-bonding. The Au-P distance ranges from 2.374 - 2.417Å in the structure, with P-Au-P angles of 128.4, 119.8 and 111.8 deg.

Figure 7, a space filling model of the structure, clearly illustrates the difficulty that large molecules would have in reaching the Au(I) center. The hydrophobic nature of this center presumably explains the inability of water molecules to quench the luminescence of the species, although this luminescence is quenched by TPA and by oxygen and other small molecules capable of electron transfer.

Fig. 7. Space filling model of $Cs_8[Au(TPPTS)_3 \cdot 5.25H_2O$,

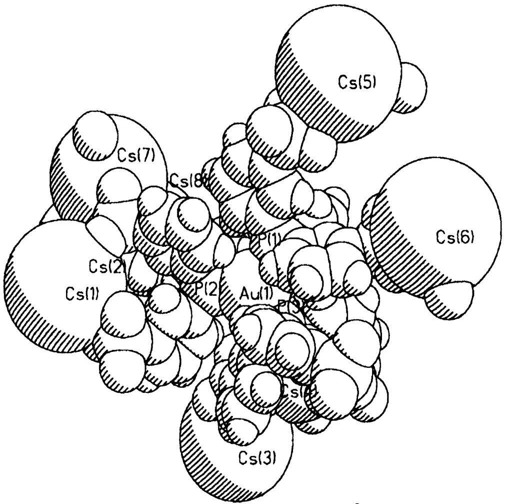

The reversible quenching of the luminescence of $[Au(TPPTS)_3]^{8-}$ by dioxygen is seen in the data presented in Figure 8. With time, the phosphine does become oxidized to O-TPPTS. This species apparently does not coordinate to the Au(I). Superoxide irreversibly quenches the luminescence, presumably due to the formation of the phosphine oxide also.

Fig. 8. Reversible emission quenching of $Na_8[Au(TPPTSD)_3]$ with dioxygen; (a) = 100% $N_2$, (b) = 100% $O_2$, (c) = 100% $N_2$.

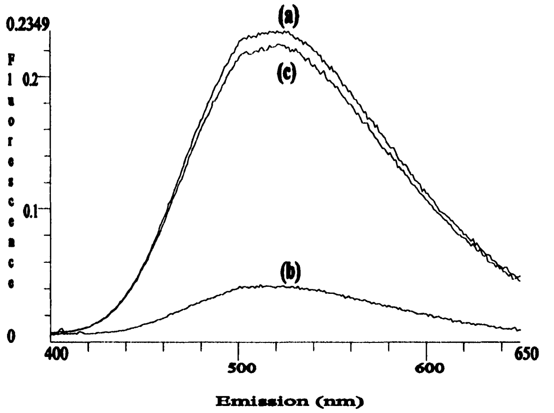

Che and Yam[24] demonstrated that the luminescent dinuclear Au(I) complex $[Au_2(dppm)_2]^{2+}$ undergoes a photo-reaction with alkylhalides in oxygen- free acetonitrile. Rate studies suggested that the likely mechanism for quenching was atom transfer with the photoexcited binuclear Au(I) complex behaving as a powerful reductant, $E^o$ ($Au_2^{3+}$ - $Au_2^{2+}$) = -1.6 V vs SSCE. Rate studies obtained by Che and Yam suggested that the C-X bond energy goverened the quenching rate. With $[Au(TPPTS)_3]^{8-}$ in $H_2O$, the correlation of quenching rate with C-X bond energies is not as clear, although the quenching rate constant from a Stern-Volmer plot for allylbromide (1.0 x $10^9$) is as order of magnitude greater than for $CCl_4$ (1.5 x $10^8$), Figure 9. The latter molecule quenches the luminescence about 25 times faster than does $CH_2Cl_2$ (5.6 x $10^6$).

Fig. 9. Quenching of $[Au(TPPTS)_3]^{8-}$ with added increments of $CCl_4$ (degassed $H_2O$ solution, 22 ± 20C, $[Au^+]$ = 10-3 M, $[CCl_4] = 10^{-3}$ M, $\lambda_{ex.}$ = 335 nm).

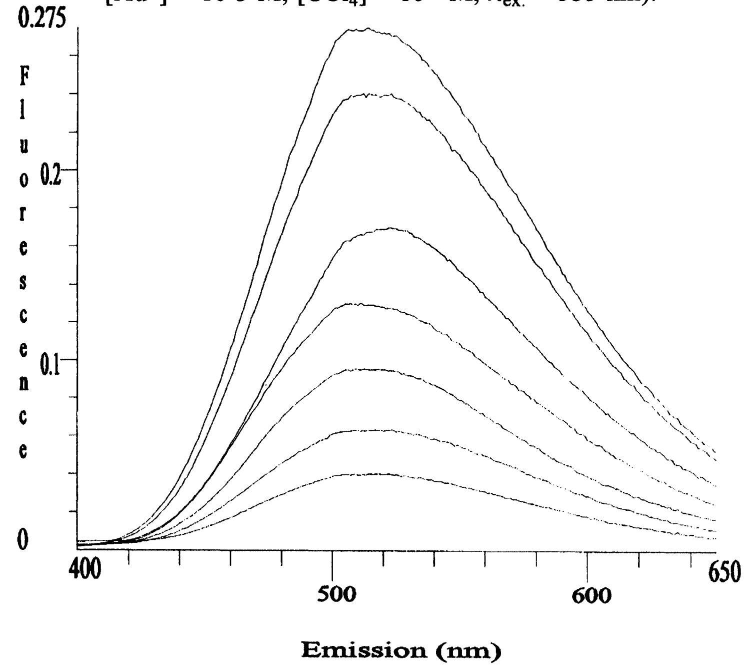

Finally, it is to be noted that the visible luminescence using a handheld UV lamp portrays a "cloudiness" suggestive of light scattering. Assuming clustering is the origin of the scattered light emitted from the water solution, ultracentrifuge measurements were undertaken to determine the apparent molecular weight of the species. These studies using a sedimentation equilibrium analytical centrifuge gave an apparent molecular wight close to that of the anion. One concludes that if the effect observed is due to scattering of the visible light by clusters, their lifetime as clusters is sufficiently short that they are not observed by this technique. In view of the rapid phosphine exchange observed by NMR, clusters large enough to scatter the very rapidly sensing visible radiation are not sufficiently well formed to influence the slow sensing sedimentation rates.

### DISCUSSION AND CONCLUSIONS

The chemical reactivity of Au(I) compounds, including active rheumatoid arthritis drugs, is related to the availability of the LUMO to obtain electrons from chemical donors or the HOMO to give up electrons by excitation. Except for direct electron transfer reactions in which the gold is reduced to the metal, not something that happens with gold drugs, the reactivity will relate to the HOMO- LUMO separation. For coordinately unsaturated complexes of Au(I), the triplet excited state (or LUMO) is only about 2 eV above the ground state (HOMO). Many Au(I) complexes show visible luminescence when the LUMO becomes the excited state by UV excitation. Metal-metal interactions and various ligands can lower the HOMO-LUMO separation, further increasing the reactivity.

The emission spectrum of the complex reflects the HOMO-LUMO energy separation in Au(I) compounds. For planar, three coordinate phosphine complexes of Au(I) such as the water soluble $[Au(TPPTS)_3]^{8-}$ anion, ultraviolet (UV) excitation produces an electronic state from which transitions to the ground state are spin and orbitally forbidden. As a result, these complexes phosphoresce with lifetimes generally in the microsecond range. Surprisingly, long-lived μs excited states even are obtained even in water. Quenching of the phosphorescence relates to the rate of reaction of substrates with the gold complex. Dioxygen, and radicals such as NO rapidly quench the excited state. However, so do oxidizing halocarbons such as $CCl_4$. Although Corey and Khan suggested that a plausible mechanism for gold drug behavior might be quenching of singlet oxygen produced in cells of RA patients, this study demonstrates that NO and $O_2^-$ react rapidly to quench excited Au(I), presumably by interacting with the LUMO of the ground state species. The cytotoxic product of the rapid reaction of superoxide with NO, peroxynitrite presumably also undergoes rapid electron transfer with Au(I) compounds, a reasonable, but to date untested, mechanism for drug action.

**ACKNOWLEDGMENTS**
Special thanks go to Richard J. Staples for providing the x-ray crystal structure of the cesium salt of TPPTS and to the Welch Foundation of Houston Texas for financial support.

**REFERENCES**

1 F. A. Cotton and G. Wilkinson, *Advanced Inorganic Chemistry*, fifth addition, Wiley Interscience, New York, 1988.
2 Jennifer M. Forword, Zerihun Assefa, Richard J. Staples, and John P. Fackler, Jr. *Inorg. Chem.*, *35*, 16- 22.(1995).
3 R. T. Sanderson, *Chemical Periodicity*, Reinhold Publishing Corp., New York, p. 34, 1960.
4 John P. Fackler, Jr., *Polyhedron,* 16(1): 1-17 (1997).
5 H. Schmidbaur, *Interdiscip. Sci. Reviews, 17*, 213-220 (1992).
6 C. Moore "Atomic Energy Levels", National Bureau of Standars, Circular 467, 1948.
7 Zerihun Assefa, Richard J. Staples, John P. Fackler, Jr., *Inorg. Chem.***33**, 2790-2798 (1994).
8 Jennifer M. Forward, Zerihun Assefa, John P. Fackler, Jr., *J. Am. Chem. Soc.,* **117**, 9103-9104 (1995).
9 Suning Wang, Guillermo Garz6n, Christopher King, Ju-Chun Wang, and John P. Fackler, Jr. *Inorg. Chem.*, *28*, 4623-4629 (1989).
10 Zerihun Assefa, Brian G. McBurnett, Richard J. Staples, John P. Fackler, Jr., Bernd Assmann, Klaus Angermaier, Hubert Schmidbaur, *Inorg. Chem.,* **34,** 75-83 (1995).
11 Zerihun Assefa, Brian G. McBurnett, Richard J. Staples, John P. Fackler, Jr., *Inorg. Chem.,* **34**, 4965-4972 (1995).
12 J. C. Vickery, M. M. Olmstead, E. Y. Fung, A. L. Balch, *Angew. Chem.,Int. Ed. Engl.,27*,417 (1997). E.Y. Fung, M. M. Olmstead, J. C. Vickery, A. L. Balch, *Coord. Chem. Rev., 171,* 151-159, (1998).
13 M. A. Mansour, W. B. Connick, R. J. Lachicotte, H. J. Gysling, R. Eisenberg, *J. Am. Chem. Soc., 120,* 1329 (1998)
14 Z. Assefa, J. P. Fackler, Jr., (to be published).
15 E. J. Coery, Mukund M. Mehrotra, Ahsan U. Khan, *Science,* **236**, 68-69 (1987).
16 Ahsan U. Khan, *J. Am. Chem. Soc,* **103**, 6516-6517 (1981).
17 David W. Abbott, Tuan Vo-Dinh, *Anal. Chem.,* **57**, 41-45 (1985).
18 J. H. Rubenspies, M.Y. Darensbourg, *Inorg. Chem.,34,*6279 (1995).
19 R. D. Scurlock, B. Wang, P. R. Ogilby, J. R. Sheats, R. L. Clough, *J. Am. Chem. Soc.,* **117**, 10194-10202 (1995).
20 J. B Lee, J. A. Hunt, J. T. Groves, *J. Am. Chem. Soc., 120,* 7493-7501 (1998).
21 Tiffany A. Grant, Ph.D. thesis, Texas A&M University, 1998.
22 Jennifer M. Forward, Zerihun Assefa, and John P. Fackler, Jr., *J. Am. Chem. Soc.*, *117*, 9103-9104 (1995).
23 Richard J. Staples collected the data and solved the structure at Harvard University, Cambridge, MA.
24 D. Li, C.-M. Che, H.-L. Kwong, V. W.-W. Yam, *J. Chem. Soc., Dalt. Trans.,* 3325 (1992).

**Received: October 10, 1998 - Accepted in final form: June 6, 1999**

